# Cerebral oxygenation and hyperthermia

**DOI:** 10.3389/fphys.2014.00092

**Published:** 2014-03-04

**Authors:** Anthony R. Bain, Shawnda A. Morrison, Philip N. Ainslie

**Affiliations:** ^1^Centre for Heart Lung and Vascular Health, University of British ColumbiaOkanagan, BC, Canada; ^2^Faculty of Professional Studies, Kinesiology, Acadia UniversityWolfville, NS, Canada

**Keywords:** hyperthermia, heat stress, cerebral blood flow, cerebral oxygenation, hemorrhage, syncope

## Abstract

Hyperthermia is associated with marked reductions in cerebral blood flow (CBF). Increased distribution of cardiac output to the periphery, increases in alveolar ventilation and resultant hypocapnia each contribute to the fall in CBF during passive hyperthermia; however, their relative contribution remains a point of contention, and probably depends on the experimental condition (e.g., posture and degree of hyperthermia). The hyperthermia-induced hyperventilatory response reduces arterial CO_2_ pressure (PaCO_2_) causing cerebral vasoconstriction and subsequent reductions in flow. During supine passive hyperthermia, the majority of recent data indicate that reductions in PaCO_2_ may be the primary, if not sole, culprit for reduced CBF. On the other hand, during more dynamic conditions (e.g., hemorrhage or orthostatic challenges), an inability to appropriately decrease peripheral vascular conductance presents a condition whereby adequate cerebral perfusion pressure may be compromised secondary to reductions in systemic blood pressure. Although studies have reported maintenance of pre-frontal cortex oxygenation (assessed by near-infrared spectroscopy) during exercise and severe heat stress, the influence of cutaneous blood flow is known to contaminate this measure. This review discusses the governing mechanisms associated with changes in CBF and oxygenation during moderate to severe (i.e., 1.0°C to 2.0°C increase in body core temperature) levels of hyperthermia. Future research directions are provided.

## Introduction

The dependence to maintain body core temperature within critically functioning limits (i.e., 37 ± 3°C) has led to seminal thermoregulatory research spanning the past 100 years (e.g., Haldane, 1905; Lindhard, 1910). From this, the capacity to effectively dissipate heat through convective and evaporative means and the concomitant cardiovascular adjustments to maintain thermoregulatory homeostasis has been topic of several extensive literature reviews (e.g., Rowell, 1974; Crandall and González-Alonso, 2010; Johnson and Proppe, 2011). Only in the last decade, however, have we begun to appropriately understand the cerebrovascular adjustments to hyperthermia. The integrative components of cerebrovascular control and ultimately oxygenation, with focus on commonly occurring levels of hyperthermia (i.e., up to +2°C core temperature) form the basis of this review. Adjustments to the three variables germane to cerebral oxygenation, fundamentally the components of the Fick equation; (1) cerebral metabolism, (2) cerebral O_2_ extraction, and (3) oxygen delivery (cerebral blood flow—CBF), are discussed. We further highlight the implications of cerebral heat balance and oxygenation during hyperthermic exercise, and provide methodological considerations for future work.

## Cerebral metabolism

The metabolic demand of human cerebral tissue is such that ~20% of total body oxygen consumption is taken up by the brain, despite only occupying 2–3% of total body mass. During passive hyperthermia of 1.5°C to 2°C above resting core temperature, whole body metabolic rate increases by ~25% (Saxton, 1981). It remains unclear whether cerebral tissue significantly contributes to the rise in whole-body metabolism during passive hyperthermia. For example, the Arrhenius activation law (or Q10, temperature coefficient), which describes the relation of biological activity to changes in temperature, implies that a rise in 2°C from 37°C should yield an increase in metabolic rate of ~10%, (South, 1958). However, the change in metabolic rate associated with the Q10 effect *in vitro* may be more sensitive during hypothermia, compared to hyperthermia (Sébert et al., 2003). Nonetheless, several animal preparations have demonstrated that local cerebral or whole-body passive heating yields an increase in cerebral glucose utilization (McCulloch et al., 1982; Mickley et al., 1997) and cerebral metabolic rate (CMRO_2_) by 5 to 10% per degree Celsius rise in core temperature (Nemoto and Frankel, 1970a,b; Carlsson et al., 1976; Busija et al., 1988). In the dog, CMRO_2_ was elevated by 21% at a rectal temperature (T_*re*_) of 42.1°C compared to baseline (T_*re*_ of 37.7); however, it began to fall at 43°C (Nemoto and Frankel, 1970b). These latter data likely reflect the temperature dependence on critical cellular activity, whereby nucleotide degradation and blood brain barrier disruption (and imminent death if not treated) begins to occur at extreme core temperatures (i.e., ≧42°C in the human) (Bynum et al., 1978). The molecular mechanisms that might impact on cerebral metabolism and oxygenation beyond a rise of 3°C have not been explored in humans, and are therefore beyond the scope of this review.

In humans, positron emission tomography measurements during passive heating to roughly +2°C rectal temperature show an increased metabolic rate of glucose in the hypothalamus, thalamus, corpus callosum, cingulate gyrus, and cerebellum (Nunneley et al., 2002). However, in the same study, significant declines in metabolic rate were observed in the caudate, putamen, insula, and posterior cingulum. To date, although regional differences are likely apparently, no study exists (to our knowledge) in the healthy awake human providing a measure of global cerebral metabolic rate during passive hyperthermia. In healthy humans during exercise, however, Nybo et al., (Nybo et al., 2002a) demonstrated with arterial and jugular venous sampling that cerebral metabolic rate is higher by ~7–8% when subjects are hyperthermic (see Discussion on Exercise). Whether the confounding factor of exercise precludes the conclusion that hyperthermia alone causes an increase in cerebral metabolism, remains unknown. Still, given the theoretical Q10 (temperature coefficient) considerations, in conjunction with animal studies, human positron emission tomography data and exercise studies, it is likely that hyperthermia (of up to +3°C) proffers a dose-dependent response to increase cerebral metabolic rate.

## Oxygen extraction

Oxygen is transported into cerebral tissue by diffusion, the speed of which is determined by the oxygen conductivity of cerebral tissue. Oxygen conductivity of cerebral tissue is fundamentally determined by the geometry of the capillaries and surrounding tissue (diffusion area and distance), and the tissue metabolism for a given oxygen gradient from the capillary to tissue (Gjedde, 2005). The speed of oxygen transport, or O_2_ extraction, can therefore be described as being inversely proportional to blood flow (when metabolism is held constant), and directly proportional to metabolism (when flow is held constant), and the surface area between the tissue and capillaries. As CBF, and subsequently O_2_ delivery is reduced, tissue extraction increases. However, because of the inverse relationship between blood flow and O_2_ extraction, when CBF is reduced by ~50–60%, the corresponding increase in O_2_ extraction (i.e., of 50–60%) is no longer sufficient to maintain a constant CMRO_2_ or adequate cerebral oxygenation (Lennox et al., 1935; Gjedde, 2005); i.e., a critical blood flow limit is reached. It follows that this theoretical critical flow limit is altered if metabolism changes; that is, the brain has a reduced critical CBF reserve for the maintenance of adequate cerebral oxygenation when metabolism (O_2_ demand) is increased. Given the above theoretical considerations, if brain metabolism increases by a liberal 10% following a 2°C increase in tissue temperature, the critical reduction in blood flow to maintain oxygenation would be ~40–50%.

## Cerebral blood flow

During passive hyperthermia, respiratory and cardiovascular adjustments blunt the coupling between CMRO_2_ and CBF. A neurogenic mechanism, i.e., cerebral vasoconstriction from increases in sympathetic nerve activity (SNA), has also been suggested to contribute to reductions in CBF during hyperthermia (e.g., Brothers et al., 2009b). Recent work in partitioning the roles of respiratory and cardiovascular mechanisms and considerations for neurogenic control of CBF during passive hyperthermia is discussed next.

### Respiratory—arterial PCO_2_ (PaCO_2_)

Hyperthermia in humans (among other species) is accompanied by a hyperventilatory response, and subsequently marked respiratory alkalosis. In 1905, Haldane was the first to describe, “breathing being more deeper and more frequent than usual” when hyperthermic (Haldane, 1905). The magnitude of the hyperventilatory response is highly variable between individuals, and is likely dependent upon the rate and magnitude of rise in skin and core temperature; however, the reflex hyperventilation is not usually pronounced until a threshold increase in core temperature of at least 1°C (Barltrop, 1954) and for review see (White, 2006). On average, a 1.5–2.0°C increase in core temperature during passive heating yields a reduction in PaCO_2_ of ~ 5–15 mmHg (see Table 1). However, the reported decline in PaCO_2_ varies considerably for a give increase in core temperature, which is likely governed by whether the external heating (i.e., skin temperature) was continued or attenuated to provide a steady-state core temperature. In some studies, PaCO_2_ can drop below 20 mmHg, and with severe passive heating (≥2°C) pronounced hyperventilation can lead to hypocapnia-induced carpopedal spasms and tetany (Iampietro et al., 1966 and unpublished observations). The exact mechanisms responsible for the hyperventilatory response during hyperthermia in humans have not been fully delineated. It is likely that a medullar integration of skin, and deep tissue temperature, principally hypothalamic temperature (Ingram and Whittow, 1962; Boden et al., 2000), primarily determine the magnitude of hyperventilatory response to hyperthermia. Temperature reception at the carotid bodies may also play an independent role (Zapata et al., 1994). For example, perfusion of warmed blood to the isolated carotid bifurcation elicits a transient hyperventilation in dogs (Bernthal and Weeks, 1939), while bilateral dissection of the carotid nerves mitigates the ventilatory increase to whole body heating in cats (Fadic et al., 1991).

**Table 1:** **Summary of human cerebral blood flow blood velocities and flow [CBF(v)] measurements during supine passive hyperthermia**.

**Authors**	**Year**	***n***	**Hyperthermia**		**ΔMAP**	**ΔPETCO2**	**ΔCBF(v)**			
			**Δcore**	**Δskin**			**ICA (%)**	**VA (%)**	**PCAv (%)**	**MCAv (%)**
Bain et al.	2013	19	+2.0°C T_es_	+5.0°C	−1 mmHg	−7 mmHg	−20	−31	−18	−23
Brothers et al.	2009a	9	+1.1°C T_gi_	+3.8°C	−1 mmHg	−4 mmHg	–	–	–	−18
Brothers et al.	2009b	7	*+1.4°C* T_gi_	+4.3°C	−1 mmHg	−6 mmHg	–	–	–	−31
Fan et al.	2008	10	+0.5°C T_es_	+3.7°C	−14 mmHg	−3 mmHg	–	–	–	−6
			+1.0°C T_es_	+3.8°C	−19 mmHg	−5 mmHg	–	–	–	−13
			+1.5°C T_es_	+4.6°C	−17 mmHg	−11 mmHg	–	–	–	−23
			+2.0°C T_es_	+4.8°C	−16 mmHg	−17 mmHg	–	–	–	−32
Low et al.	2008	9	+1.1°C T_gi_	+3.7°C	−2 mmHg	−3 mmHg	–	–	–	−13
Nelson et al.	2011	10	+0.9°C T_gi_	+3.5°C	0 mmHg	−2 mmHg	–	–	−10	−7
		8	+1.8°C T_gi_	+5.8°C	−2 mmHg	−15 mmHg	–	–	−23	−26
Ogoh et al.	2013	12	+0.3°C T_es_	+3.8°C	−1 mmHg	−2 mmHg	−5	−8	–	−15
			+0.7°C T_es_	+4.7°C	−4 mmHg	−2 mmHg	−5	−9	–	−15
			+1.2°C T_es_	+5.1°C	−3 mmHg	−5 mmHg	−12	−12	–	−26
			+1.4°C T_es_	+5.1°C	−6 mmHg	−6 mmHg	−18	−17	–	−23
Wilson et al.	2006	15	+0.9°C T_gi_	+4.2°C	0 mmHg	−2 mmHg	–	–	–	−15^*^

Asterisks (

* *) indicate values estimated from figure representation. T_*re*_, T_es_, and T_gi_ represent rectal, esophageal, and gastrointestinal temperature respectively. n = sample size. MAP = mean arterial pressure. P_ET_CO_*2*_ = end-tidal CO_*2*_ partial pressure. ICA = Internal carotid artery blood flow. VA = vertebral artery blood flow. MCAv = middle cerebral artery blood velocity. PCAv = posterior artery blood velocity*.

**Figure 1:**
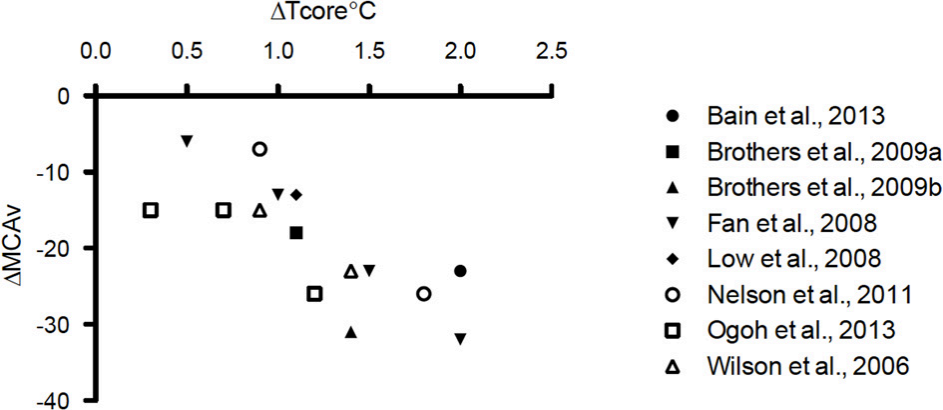
**Representation of the reported percent reductions in middle cerebral artery blood velocity (MCAv) (x axis) as a function of delta core temperature (esophageal, gastrointestinal, or rectal) (y axis) during supine passive hyperthermia up to +2°C**.

**Figure 2:**
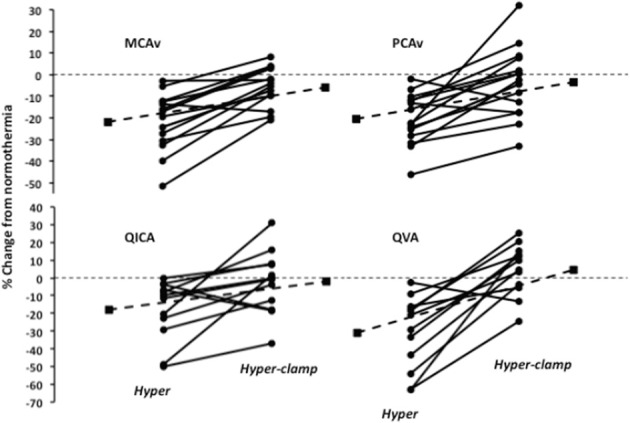
**Change in middle cerebral artery blood velocity (MCAv), posterior cerebral artery blood velocity (PCAv), internal carotid artery blood flow (QICA) and vertebral artery blood flow (QVA) following a 2.0°C rise in esophageal temperature with and without restoration of end-tidal CO_2_**. Adapted from (Bain et al., 2013).

It is well established that PaCO_2_ is a potent modulator of CBF (Ainslie and Duffin, 2009). At rest, each mmHg change in PaCO_2_ above and below eupnia yields an approximate 4% increase and 2% decrease in CBF, respectively (Willie et al., 2012; and Willie et al., 2014 for review). During passive supine hyperthermia of +1–1.5°C core temperature above resting, a 10–20% reduction in cerebral blood flow is typically observed (see Figure 1 and Table 1). The role of PaCO_2_ in the reduction of CBF during hyperthermia remains debatable. Bain et al. (2013) recently demonstrated, using both volumetric and intra-cranial velocity measurements, that global (anterior and posterior) CBF during supine severe (+2°C esophageal temperature) hyperthermia is completely restored to normothermic values upon returning end-tidal CO_2_ (P_*ET*_CO_2_) back to normothermic levels (Figure 2). This finding is principally corroborated by other studies (Fan et al., 2008; Nelson et al., 2011). It should be noted, however, that although middle cerebral artery (MCAv) and posterior cerebral artery (PCAv) velocities were statistically restored to normothermic values following P_*ET*_CO_2_ restoration during +2°C hyperthermia in Nelson et al. (2011), they were still 9 and 3% lower respectively, than baseline values. To that end, in opposition of complete CBF restoration following a return to eucapnia, Brothers et al. (2009b) reported that MCAv was only 50% restored back to normothermic values upon the restoration of P_*ET*_CO_2_ during supine hyperthermia. Although difficult to reconcile, these divergent findings may be explained by the variability in “steady-state” CBF following baseline P_*ET*_CO_2_ restoration. For example, although absolute CO_2_ reactivity appears to be maintained during hyperthermia (Low et al., 2008), the dynamics of the CBF response to eucapnic restoration will in part be determined by the magnitude of the hyperventilation response (and resultant respiratory alkalosis) (Ide et al., 2003). That is, those with a larger hyperventilatory response will likely require a longer time to reach steady-state CBF values upon restoration of baseline PaCO_2_ due to a larger extra-cellular pH gradient. Moreover, sustained hypoventilation may eventually lead to an adaptive response whereby restoration of baseline eucapnia will yield a temporary overshoot in CBF (compared to pre-stimulus values) (Ide et al., 2003). The mechanisms of this CBF overshoot remains unclear, but may involve changes in lactate and bicarbonate (Albrecht et al., 1987; Marder et al., 1990). As such, the transient magnitude of the CBF overshoot probably depends upon the length of time and magnitude of the hypocapnia. Given the above, it is clear that the magnitude and duration of hypocapnia can influence “steady-state eucapnic” CBF measures, and may explain the variable conclusions for the role of PaCO_2_ in reducing CBF during hyperthermia. Nonetheless, taken the most recent data (Nelson et al., 2011; Bain et al., 2013), it is more than likely that PaCO_2_ explains the majority of the CBF reduction during passive hyperthermia, at least when subjects are kept in the supine position. Still, future research is warranted to better clarify this role.

In contrast to supine hyperthermia, during upright seated hyperthermia, Fujii et al. (2008) and Ross et al. (2012), found that MCAv is only partially restored back to normothermic levels upon restoration of P_*ET*_CO_2_ with the addition of 5% CO_2_ to the inspired air. Furthermore, Nelson et al. (2011) found that head up tilt exacerbated the decline in MCAv and PCAv while hyperthermic, in the absence of significant further reductions in P_*ET*_CO_2_. It is therefore evident that, during hyperthermia, CBF is declined by increased hydrostatic pressure associated with posture (see Cardiovascular Section), independently of PaCO_2_.

#### Do changes in PaCO_2_ alter tolerance time to a simulated hemorrhage?

Tolerance time to a simulated hemorrhage is clearly reduced while hyperthermic compared to normothermic (Allan and Crossley, 1972; Wilson et al., 2006; Keller et al., 2009; Brothers et al., 2011b). Reductions in PaCO_2_ associated with hyperthermia-induced hyperventilation appear to have little influence on the reduced ability to withstand simulated hemorrhage (Lucas et al., 2013; Pearson et al., 2013). This suggests that cardiovascular adjustments contribute more to tolerance time (i.e., minimum cerebral oxygenation levels before syncope) than baseline CBF during a graded hemorrhage simulation. This notion is supported by findings from our laboratory where tolerance to graded lower body negative pressure was unaltered even when baseline CBF was reduced by ~30% via administration of indomethacin, independently of changes in PaCO_2_ (Lewis et al., under review). Such findings may be attributed to the fact that simulated hemorrhage time is typically determined by the time elapsed before ethically low blood pressure levels (usually a SBP of <80 mmHg) are attained, rather than syncope itself. A perhaps more ecological stance is the view that a reduction in CBF at baseline, although not effecting tolerance time to simulated hemorrhage, effectively reduces the buffer zone for CBF to change before syncope occurs. As such, when PaCO_2_ and subsequently CBF, is reduced from hyperthermia, any condition eliciting a faster or larger perturbation in BP (i.e., a period when cerebral autoregulation is less effective) (Tzeng and Ainslie, 2013) compared to graded lower body negative pressure, may pose an increased risk of syncope. It should be noted, however, that dynamic cerebral autoregulation, as indexed by steady-state linear transfer function analysis, appears to be maintained (Low et al., 2009) or perhaps even improved, with hyperthermia (Brothers et al., 2009a).

### Cardiovascular control

In order to promote heat loss via evaporative and convective means during severe passive hyperthermia, cutaneous blood flow can increase upwards of 25-fold (e.g., from ~300 to 7500 mL·min^−1^) (Rowell et al., 1969; Rowell, 1986). The large increase in cutaneous vascular conductance is met by concomitant increases in cardiac output (at times up to 13 mL·min^−1^) (Rowell et al., 1969; Rowell, 1986), accomplished almost exclusively via increases in heart rate. In turn, it is now well accepted that resting BP, and therefore perfusion pressure to the brain during passive, supine hyperthermia, is generally preserved, or only moderately decreased (see Crandall and González-Alonso, 2010 for a comprehensive review on the cardiovascular functioning during hyperthermia). It is interesting to note, however, that BP estimations during passive hyperthermia vary considerably (see Table 1). These variations likely reflect the difficulty in acquiring accurate BP measurements without measuring it intra-arterially during hyperthermia (Ganio et al., 2011). Nonetheless, in contrast to passive supine hyperthermia, it is generally accepted that adequate BP is not maintained under dynamic hyperthermic conditions, e.g., with an orthostatic challenge or hemorrhage.

Any condition that compromises CBF maintenance inherently increases the risk of syncope/reduction of cerebral oxygenation. As mentioned, tolerance to an orthostatic challenge or simulated hemorrhage is reduced when hyperthermic (Allan and Crossley, 1972; Wilson et al., 2006; Keller et al., 2009; Brothers et al., 2011b). Given that changes PaCO_2_ seem to play a negligible role in determining tolerance time to a simulated hemorrhage (see section *Do changes in PaCO_2_ alter tolerance time to a simulated hemorrhage?*), two key cardiovascular adjustments are likely responsible; (1) the inability to decrease systemic vascular compliance (SVC) (Wilson et al., 2002a; Ganio et al., 2012), and (2) a greater reduction in stroke volume for a given reduction in left ventricular filling pressure (i.e., a leftward shift of the operating point to a steeper portion on the Frank Starling curve) (Wilson et al., 2009). Clearly, the former dictates the latter. When normothermic, it is well established that SVC decreases during a simulated hemorrhage (Murray et al., 1968). Why SVC does not also decrease when hyperthermic, is not entirely understood. However, it is generally accepted that an inhibition of cutaneous vasoconstriction is likely at play (Crandall et al., 2010). An improvement to orthostatic tolerance following acute skin cooling while hyperthermic lends evidence to this hypothesis (Wilson et al., 2002b). The mechanisms of cutaneous vasculature control remains a complex field of study, riddled with redundant mechanistic pathways (for a review see Charkoudian, 2010). Nonetheless, it appears that human physiology places a hierarchy for heat loss during hyperthermia, potentially to the detriment of adequate central blood volume and subsequently CBF/consciousness.

Dehydration (i.e., ≥2% loss of body mass) often follows prolonged sweating, and is therefore closely tied to hyperthermia. A major cardiovascular consequence of dehydration is a dose-dependent decrease in blood volume (Kempton et al., 2009). In turn, dehydration impairs the ability to maintain adequate central blood volumes, and thus CBF during an orthostatic challenge (Harrison et al., 1986; Romero et al., 2011). Carter et al. (2006) demonstrated that the transient reductions in MCAv were larger upon standing from sitting when dehydrated (3.0% reduction in body mass), compared to euhydrated. Consistent with this finding, Moralez et al. (2012) demonstrated that dehydration (2.7% reduction in body mass) exacerbated the reductions in BP and MCAv upon standing following a 10-rep maximum leg press. It is therefore reasonable to assume that when hyperthermia is coupled with dehydration, the ability to maintain adequate CBF is further reduced during orthostatic challenges or hemorrhage.

In contrast to the apparent reduction in CBF with an orthostatic challenge when dehydrated, Fan et al., (Fan et al., 2008) demonstrated that when subjects were supine, dehydration (1.5% reduction in body mass) increased resting MCAv by ~11%. When subjects were made hyperthermic, however, dehydration appeared to have little or no effect on the reduction in MCAv. It is difficult to reconcile why MCAv (and blood flow in the common carotid artery) was increased with normothermic dehydration compared to euhydration. Increases in CBF during passive supine dehydration may be related to the increased osmolality of extracellular fluid via cerebral cellular shrinkage (i.e., increased concentrations of solutes in the extracellular fluid cause an intra-to extra cellular fluid shift (Kempton et al., 2009). In turn, CBF during supine dehydration may be increased to maintain an appropriate ionic milieu for neuronal function. Nonetheless, during passive supine hyperthermia, the marked reductions in MCAv associated with the reduced PaCO_2_ seem to shadow any effect of dehydration (Fan et al., 2008).

### Neurogenic control

Sympathetic nerve activity in the muscle and skin vasculature is significantly elevated during hyperthermia (Bini et al., 1980; Niimi et al., 1997; Cui et al., 2004; Keller et al., 2006). Hyperthermia decreases vascular conductance of the splanchnic and renal tissue, presumably also via increased SNA (Rowell, 1983). Indeed, it is commonly accepted that the primary mechanism of blood flow redistribution to the cutaneous tissue during hyperthermia is driven by SNA (Rowell, 1990). Whether increased SNA during hyperthermia affects the cerebral vasculature, however, remains speculative. It is well recognized that perivascular adrenergic nerves richly innervate the cerebral arteries, (Edvinsson and Hamel, 2002), while the smooth muscle cells of the arterioles possess both alpha- and beta-adrenergic receptors (Edvinsson, 1982). This suggests that the cerebral vascular has the potential to be mediated by neurogenic factors. In animal models, CBF is reduced with stimulation of the superior cervical ganglion (Heistad et al., 1978; Cassaglia et al., 2008). In humans, unilateral trigeminal ganglion stimulation decreases CBF (Visocchi et al., 1996), while stellate ganglionic blockade increases CBF (Umeyama et al., 1995; Ide et al., 2000). The above animal and human studies support a tonic neurogenic control of CBF. Therefore, several authors have speculated that reductions in CBF during hyperthermia may, in part, be due to increases in cerebral SNA (see Crandall and González-Alonso, 2010 and related references). However, this notion is based primarily by deduction when PaCO_2_ and MAP cannot explain the full reduction in CBF (see Discussion on the Role of Arterial PCO_2_). Although an attractive hypothesis, several caveats persist to accept that SNA decreases CBF during hyperthermia. First, redundant mechanisms (e.g., dilator agents such as nitric oxide, prostanoids, and histamine) may act to counteract a noradrenaline-induced vasoconstriction of the cerebral vasculature. Specifically, when brain metabolism is elevated (see Discussion on Metabolism and Hyperthermia), a “functional sympatholysis” or “metabolic restraint” might mitigate the influence of SNA (Gross et al., 1983; Busija and Leffler, 1987). This is in agreement with animal studies that report a global increase in CBF to passive hyperthermia (Carlsson et al., 1976; Busija et al., 1988) that cannot be entirely explained by changes in PaCO_2_. Second, the density of alpha- and beta-adrenergic receptors on the cerebral arterioles varies depending on vessel size (Edvinsson, 1982), suggesting that a heterogeneous response, potentially modified by hyperthermia, may exists for a given increase in SNA. Third, the relative influence of SNA on the cerebral vascular seems to be dependent upon levels of blood pressure. That is, sympathetic activation has a larger influence during hypertension, compared to normotension (Bill and Linder, 1976; Edvinsson et al., 1976; and reviewed in Willie et al., 2014). Lastly, the cerebral vasculature has been shown to exhibit a “vasomotor escape” following prolonged maximal SNA stimulation of over 5 ± 7 min (Sercobe et al., 1979), suggesting that the influence of SNA on the cerebral vasculature may be dependent on the duration of the stimulation. That CBF has recently been shown to fully recover to normothermic values when PaCO_2_ is returned to eucapnia (Bain et al., 2013) supports the notion that increases in SNA during hyperthermia proffer a negligible effect on global CBF. Nonetheless, future studies are required to better understand this potential mechanism. Administration of a centrally acting α2-adrenoreceptor agonist (provided no changes in MAP), cervical ganglion block, or measurements of cerebral noradrenaline spillover (Mitchell et al., 2009) during hyperthermia with concomitant measures of global CBF and maintenance of eupnia, is likely warranted.

## Cerebral heat balance, oxygenation, and exercise

Human cerebral tissue uses oxygen at a metabolic rate of between 3 to 3.5 mLO_2_· 100 g cerebral tissue^−1^·min^−1^ (Lassen, 1985), producing approximately 0.6 j g^−1^ · min^−1^ heat, which must then be removed via cerebral circulation (reviewed in: Nybo and Secher, 2004). During hyperthermia, cerebral heat balance is compromised from reductions in CBF (Nybo et al., 2002b) and therefore reductions in convective heat loss (arguably the only avenue for cerebral heat loss). An increased cerebral temperature can impair blood-brain barrier integrity (Watson et al., 2005), particularly when combined with dehydration (Watson et al., 2006). The exact interaction between temperature, blood brain barrier opening and cerebral oxygenation remains obscure.

During hyperthermia exercise capacity is reduced (e.g., Rowell et al., 1966, and for reviews of the potential mechanisms involved see Nybo, 2007 and Cheuvront et al., 2010). Reductions in exercise capacity and a sooner onset of fatigue likely stem from interactions of both central and peripheral factors (Nybo and Secher, 2004), including observed alterations in EEG α/β arousal levels with greater perceived exertion and decreased motor unit activation (Nybo and Nielsen, 2001; Morrison et al., 2004; Todd et al., 2005; Périard et al., 2011). Decreases in voluntary activation tend to correlate to reductions in MCAv (measured via Doppler ultrasound), but these reductions can be partially restored when breathing a hypercapnic gas mixture to offset changes in ventilation and P_*ET*_CO_2_ levels from heat-induced hyperventilation (Ross et al., 2012). However, although preventing hypocapnia during normothermic exhaustive cycling exercise can exhibit increases in MCAv, performance is unchanged (Subudhi et al., 2011). As such, it is likely that a direct effect of increased temperature on CNS and neuromuscular functioning, rather than detriments to cerebral oxygenation, is the primary factor governing decreased exercise capacity while hyperthermic.

Examinations of cerebral oxygenation during exercise using near-infrared spectroscopy (NIRS) suggest that cerebral oxygenation is not impaired, including when subjects are passively heated to core temperatures up to 39.5°C (Morrison et al., 2009). However, it is now clear that changes in skin blood flow can alter the NIRS-derived oxygenation values (e.g., Davis et al., 2006); thus, data using only this measure must be interpreted with caution. Using the Kety-Schmidt protocol to measure global CBF, Nybo et al., (2002a) and Rasmussen et al. (2010) reported that uncompensable hyperthermic exercise elicited reductions in CBF by ~18 and 15% greater than “normothermic” exercise respectively. Of note, Rasmussen et al. (2010) further estimated cerebral mitochondrial oxygen tension, and found it to be declined by ~5 mmHg during hyperthermic compared to normothermic exercise. This reduction was attributed to the fact that cerebral metabolic rate of oxygen increased by 8 ± 7% from the beginning to the end of hyperthermic exercise, while CBF decreased by 15%, and O_2_ extraction only increased by 7% (Rasmussen et al., 2010). Given these data, it can be rationalized that cerebral oxygenation is in fact compromised during exhaustive hyperthermic exercise. However, the finding that O_2_ extraction did not increase sufficiently to maintain mitochondrial oxygenation in the face of moderate increase in metabolic demand (8%) and reduction in CBF (15%), is in contradiction to theoretical considerations (see Oxygen Extraction section). Furthermore, the estimations of mitochondrial oxygen tension are inherently based on several assumptions. For example, the diffusibility of O_2_ must remain constant (Rasmussen et al., 2007) [supported by the lack of capillary recruitment in rats during hypoxia (Göbel et al., 1989)]; and the potential for cerebral oxygen stores via *neuroglobin* (Burmester et al., 2000) to preserve mitochondrial oxygenation when O_2_ availability declines, are also ignored. Nonetheless, these calculations provide the best estimations of cerebral mitochondrial oxygen tension to date in humans. However, it remains that a consensus on global cerebral oxygenation during hyperthermic exercise is difficult to ascertain, and requires further experimentation.

## Conclusions and future directions

**Figure 3:**
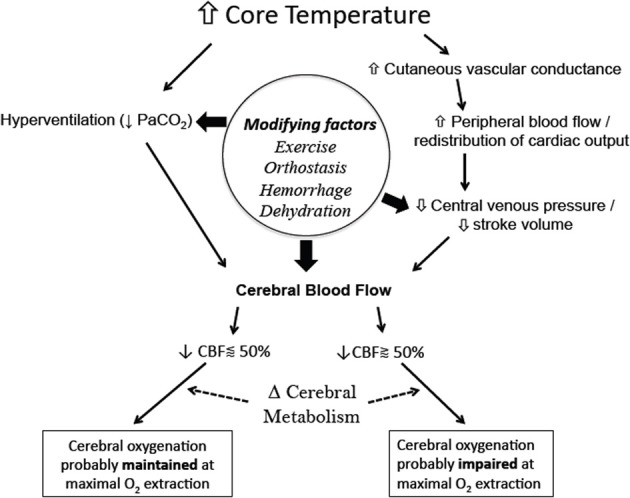
**Simplified schematic of the mechanisms and modifying factors involved with reductions in cerebral blood flow and ultimately cerebral oxygenation during whole-body hyperthermia**. Global cerebral oxygenation is likely impaired when CBF is reduced beyond 50%, i.e., a critical blood flow is reached at maximal levels of oxygen extraction. Changes in cerebral metabolism will alter the theoretical critical blood flow limit, while regional changes in metabolism and blood flow can yield regional differences in cerebral oxygenation.

The fate of cerebral oxygenation during hyperthermia of up to +2°C core temperature is dependent upon the integrative balance between increases in metabolism and oxygen extraction, with declines in cerebral perfusion pressure from reductions in PaCO_2_ and increased systemic vascular conductance (Figure 3). When left in the supine position, a ~10 mmHg drop in PaCO_2_ following a 2°C increase in core temperature yields an average CBF reduction by ~25%. At which point, it stands to reason that the global theoretical capacity to increase cerebral O_2_ extraction is, on average, effective in maintaining cerebral oxygenation, even with an increase in cerebral metabolism of ~10%. On the other hand, the inability of the cardiovascular system to maintain perfusion pressure to the brain during more dynamic conditions (e.g., hemorrhage or orthostatic challenge), coupled with a reduced CBF baseline from reductions in PaCO_2_, potentiates a condition whereby cerebral oxygenation could be compromised following maximal O_2_ extraction potential. This fact is clearly evidenced by the reduced tolerance time to simulated hemorrhage, and the increased occurrence of syncope during hyperthermia.

Recent data have collectively provided a salient understanding of cerebral oxygenation during varying degrees of whole-body hyperthermia, however several avenues of experimentation remain. First, it is evident that direct measurements of arterial and cerebral venous blood in humans are required to experimentally verify changes in cerebral metabolism and oxygenation with separate levels of CBF during hyperthermia. Second, albeit inherently difficult to execute, a conclusive study on the role of SNA on CBF during hyperthermia is required. Third, the importance of extra-cranial contamination on NIRS-derived oxygenation values has been highlighted during changes in skin blood flow (Davis et al., 2006) and also where scalp ischemia induced by inflation of a circumferential cranial tourniquet impacted NIRS readings (Davie and Grocott, 2012). Although newer clinically available NIRS monitors use algorithms to subtract light absorption from superficial tissue (e.g., scalp, skin, bone, pia matter) from deeper tissue (Zheng et al., 2013), the utility during hyperthermia and/or exercise remains to be established. Lastly, the interactive role of dehydration, heat acclimatization and certain pathologies (e.g., heart failure, diabetes, autonomic disorders, etc.) on cerebral oxygenation during heat stress should be focus for future work.

### Conflict of interest statement

The authors declare that the research was conducted in the absence of any commercial or financial relationships that could be construed as a potential conflict of interest.
